# Corrosion Investigation of Reinforced Concrete Based on Piezoelectric Smart Materials

**DOI:** 10.3390/ma12030519

**Published:** 2019-02-09

**Authors:** Weihua Shi, Ying Chen, Peng Liu, Dongyu Xu

**Affiliations:** 1 Hunan Provincial Key Laboratory of Structures for Wind Resistance and Vibration Control & School of Civil Engineering, Hunan University of Science and Technology, Xiangtan 411201, China; llppp98@163.com; 2 School of Civil Engineering, Central South University of Forestry and Technology, Changsha 410075, China; 3 School of Civil Engineering, Central South University, Changsha 410075, China; liupeng868@csu.edu.cn; 4 Shandong Provincial Key Laboratory of Construction Materials Preparation and Measurement, University of Jinan, Jinan 250022, China; mse_xudy@ujn.edu.cn

**Keywords:** reinforced concrete, piezoelectric, electromechanical, impedance, corrosion

## Abstract

An embedded piezoelectric transducer was developed for monitoring the corrosion process of reinforcement bars in concrete based on the piezoelectric impedance technique. The electrochemical method was employed to accelerate the corrosion process of the reinforcement bar with relative mass loss of 0.5–10%, and the resistance spectra of the piezoelectric transducers were investigated to assess the corrosion process. The results show that the corrosion process of the reinforcement bar has significant influence on the resistance spectra of the piezoelectric transducers. Statistical parameters were used to intuitively evaluate the corrosion evolution based on variations of the resistance spectra. The corrosion process of reinforcement bar in concrete can be classified into three periods; that is, the initial period when the relative mass loss is less than 2%, the developing period at a relative mass loss of 2–4%, and the rapid corrosion period when the relative mass loss is higher than 4%.

## 1. Introduction

Concrete is presently a widely utilized construction material in the field of civil engineering, and still will be the most important construction material in the future. However, the concrete structures in service will inevitably be destroyed due to effects of various environmental and natural disasters, thus leading to significant casualties and economic losses. Corrosion of reinforcement bars is one of the most important factors that causes the invalidation of concrete structures [1,2], especially in some coastal construction projects, such as ports, long-span bridges, and offshore drilling platforms. The structural disasters due to corrosion of reinforcement bars in concrete, such as the decrease of structural bearing capacity, expansion cracking of concrete, and deterioration of bonding ability, also appear frequently throughout the world, which has caused extensive concern [3,4,5]. It is reported that serious steel corrosion exists in the coastal concrete engineering that was constructed before 1990s in Chia. Therefore, it is an important issue to perform online monitoring on these critical concrete structures to ensure their reliable service.

The corrosion process of reinforcement bars in concrete is an electrochemical process. The passivation film of a reinforcement bar is destroyed by incursion of the external surroundings, and a chemical battery reaction on the steel is accordingly commenced under surroundings of water and oxygen. Effective data are needed to predict the residual life of concrete structures through monitoring the corrosion process of reinforcement bars in concrete. Existing corrosion monitoring techniques can be categorized into analytical methods, physical methods, and electrochemical methods. The analytical methods [6,7,8] determine the corrosion extent of reinforcement bars in concrete by building mathematical models in terms of variation of dimension and thickness of the protective layer of the reinforcement bar, concrete strength, and longitudinal crack width, etc. The physical methods employ some advanced materials and facilities to determine the corrosion process by monitoring the physical phenomena in the corrosion process, such as resistance and electromagnetic parameters [9,10] and acoustic and optical wave [11,12,13]. The electrochemical methods show great potential in monitoring of relative mass loss and extent of reinforcement bars in concrete through variation of electrochemical parameters in the corrosion process [14,15,16]. Recently, with rapid development of smart materials and structures in the field of civil engineering, some smart materials which are well-used in the fields of aerospace and mechanical engineering have also been transplanted into concrete engineering [17,18,19,20,21,22,23]. Among them, piezoelectric materials have gained particular attention due to their merits of fast response time, simple structure, and good reliability. Some online monitoring methods based on piezoelectric transducers have also been developed, such as the piezoelectric strain technique, ultrasonic technique, and piezoelectric impedance technique [17,24,25,26].

It is known that the mechanical characteristics of reinforcement bars in concrete, such as stiffness and damping, will change when suffering from corrosion. If piezoelectric transducers are coupled with concrete, the mechanical impedance of the corroded reinforcement bar will affect the electric impedance versus frequency spectra of the piezoelectric transducers [27,28]. Therefore, a kind of piezoelectric impedance technique was proposed in this study to monitor the corrosion process of reinforcement bars in concrete by developing a kind of embedded piezoelectric transducer.

## 2. Experiments

### 2.1. Principle of the Piezoelectric Impedance Technique

**Figure 1:**
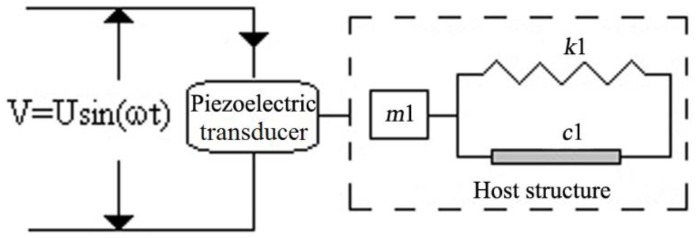
One-dimensional interaction model of the piezoelectric transducer and host structure. V: the instantaneous voltage; U: maximum value of voltage; ω: angular frequency; t: time; c1: damper; k1: spring; m1: mass.

Figure 1 shows the one-dimensional interaction model between the piezoelectric transducer and host structure, in which the host structure can be regarded as a system of the spring (*k*1)-mass (*m*1)-damper (*c*1) model. 

It is known based on the theoretical model proposed by Liang et al. [24] that the electrical admittance *Y(ω)* of a piezoelectric transducer is a combined function of the mechanical impedance Z_a_(*ω*) of piezoelectric transducer and the Z(*ω*) of the host structure:

(1) $$Y(\omega )=\frac{I}{V}=i\omega a(\varepsilon_{33}^{T}-\frac{Z(\omega )}{Z(\omega )+Z_{a}(\omega )}d_{3x}^{2}Y_{xx}^{E})$$

where *V* is the input voltage of piezoelectric transducer, *I* is the output current from the transducer, and *a*, *d*_3x_, $Y_{xx}^{E}$, and $\varepsilon_{33}^{T}$ are the geometry constant, piezoelectric coupling constant, complex Young’s modulus at zero electric field, and complex dielectric constant of the piezoelectric transducer at zero stress, respectively. 

The electrical impedance of a piezoelectric transducer is related to the mechanical impedance of the host structure in this equation, and any changes of electrical impedance of the piezoelectric transducers can be considered as an indication of the structural integrity. Thus, the damage condition of the host structure can be monitored by analyzing the variation of electric impedance of the piezoelectric transducer. 

### 2.2. Corrosion Experiment of the Reinforcement Bar in Concrete

**Figure 2:**
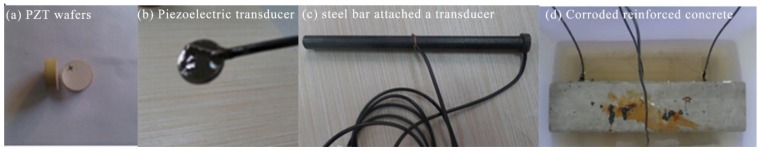
Photographs of the piezoelectric transducer and corroded reinforced concrete. (**a**) PZT (Lead Zirconate Titanate) wafers; (**b**) Piezoelectric transducers; (**c**) Steel bar attached a transducer; (**d**) Corroded reinforced concrete.

Two PZT (Lead Zirconate Titanate) wafers of Φ 12 × 2 mm^2^ (#1) and Φ 12 × 5 mm^2^ (#2) were used to fabricate the piezoelectric transducers, as shown in Figure 2a. First, the shielding wires were welded to both electrodes of the wafers, and then cement, epoxy resin, and hardening agent were mixed together to encapsulate the PZT wafers with a mass ratio of 1:1:0.25. Before packaging, the mixture was first put into the vacuum chamber for 5 min to reduce the air pores, and a thin layer of the mixture of 1 ± 0.2 mm in thickness was then coated on both PZT wafers and the exposed parts of the wires, as shown in Figure 2b. After solidifying of the packaging layer, the PZT transducers were tested in water to ensure their reliability in a practical monitoring application.

**Figure 3:**
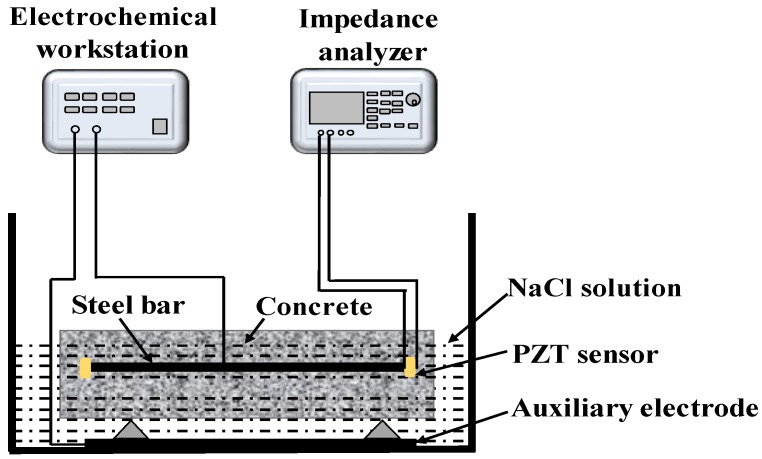
Schematic diagram of corrosion monitoring of reinforced concrete.

Here, a round steel bar of Φ 14 × 340 mm^2^ was monitored, and the mixture of cement, epoxy resin, and hardening agent mentioned above was used to stick PZT transducers to both ends of the steel bar. In addition, a wire was also welded to the middle part of the steel bar to connect the electrochemical workstation, as shown in Figure 2c. The C30 concrete was designed with a mass ratio of cement/sand/stone/water of 1:2.2:3.6:0.6. The corrosion process of steel in a natural environment is a slow process; therefore, the accelerated corrosion of reinforced concrete was performed by using the electrochemical workstation, as shown in Figure 3. The concrete block was placed in a container with 5% NaCl solution. Stainless steel was used as the auxiliary electrode of the electrochemical workstation, and the reinforcement bar in concrete was used as working electrode. The current density was maintained at 1 mA cm^−2^. The impedance analyzer was employed to acquire the electric impedance data of PZT transducers.

Based on the electrochemical corrosion principle, it is known that an oxidation–reduction reaction exists in the electrochemical corrosion process of steel. The reaction is usually expressed as follows:

Fe(s) − 2e^−^ = Fe^2+^ (aq) (2)

O_2_(g) + 2H_2_O(aq) + 4e^−^ = 4OH^−^ (aq) (3)

The relative mass loss of steel can be controlled by changing the current intensity, and the corrosion mass of steel can then be obtained theoretically according to Faraday’s laws, as shown in Equation (4). The relative mass loss (Δν) was calculated based on the mass loss rate of reinforcement bar before and after corrosion, as shown in Equation (5).

(4) $$\Delta m=n\cdot M=\frac{Q\cdot M}{F\cdot \left|Z\right|}=\frac{M\int I(t)dt}{F\cdot \left|Z\right|}$$

(5) $$\Delta \upsilon =\frac{\Delta m}{m}$$

where Δ*m* is the mass of steel being corroded, *m* is the mass of steel before corrosion, *Q* is the quantity of electric charge, *F* is the Faraday constant, *I*(*t*) and *t* are the current intensity and time, |Z| is the absolute value of valence of ferric iron, *n* is the amount of substance of the corroded steel, and M is the molar mass of iron.

### 2.3. Electric Impedance Test of PZT Transducers

The electric impedance spectra of PZT transducers were recorded by the precision impedance analyzer. According to the monitoring principle of the piezoelectric impedance technique, the variation of mechanical impedance of steel in concrete can be obtained by analyzing the electric impedance variation of PZT transducers. Here, concrete cured in standard conditions (temperature: 20 ± 2 °C, relative humidity: ≥95%) for 28 days was regarded as the pristine state of the corrosion process, and the electric impedance value at this state was used as baseline. The electric impedance spectra of PZT transducers were tested in a frequency range of 40–800 kHz. In order to improve the monitoring resolution, the tested frequency range was divided into 32 frequency intervals, and there were 801 frequency points in every frequency interval. The electrochemical workstation was employed to control the current intensity and duration of polarization based on Faraday’s laws, and the electric impedance data were recorded every 0.5% until a relative mass loss of 10% was reached.

## 3. Results and Discussion

### 3.1. Impedance Spectra Comparison of PZT Transducers before and after Packaging

It is well known that piezoelectric materials vibrate under excitation of alternating current due to the converse piezoelectric effect, and the resonant phenomena can usually be observed from the impedance spectra. According to the principle of the piezoelectric impedance technique, the impedance spectra of PZT transducers with packaging layer will be different from that without the packaging layer; therefore, the mechanical impedance variation of the coupled structure can be indicated based on this impedance variation of PZT transducers.

**Figure 4:**
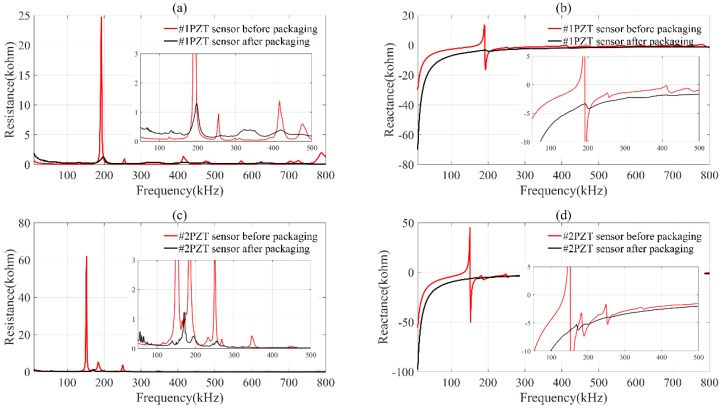
The resistance and reactance spectra of PZT transducers before and after packaging. (**a**) resistance spectra of 1#PZT sensor before and after packaging; (**b**) reactance spectra of 1#PZT sensor before and after packaging; (**c**) resistance spectra of 2#PZT sensor before and after packaging; (**d**) reactance spectra of 2#PZT sensor before and after packaging.

Figure 4 presents the resistance and reactance of PZT transducers before and after packaging. There exist a series of resonant peaks in a frequency range of 40–800 kHz, especially in the resistance spectra. The frequencies of the dominant resonant peaks are about 200 kHz for PZT transducer #1 and 180 kHz for PZT transducer #2. After packaging, it can be clearly observed that the amplitude of the resonant peaks decreases sharply and some weak resonant peaks even disappear, while some new resonant peaks also appear.

**Figure 5:**
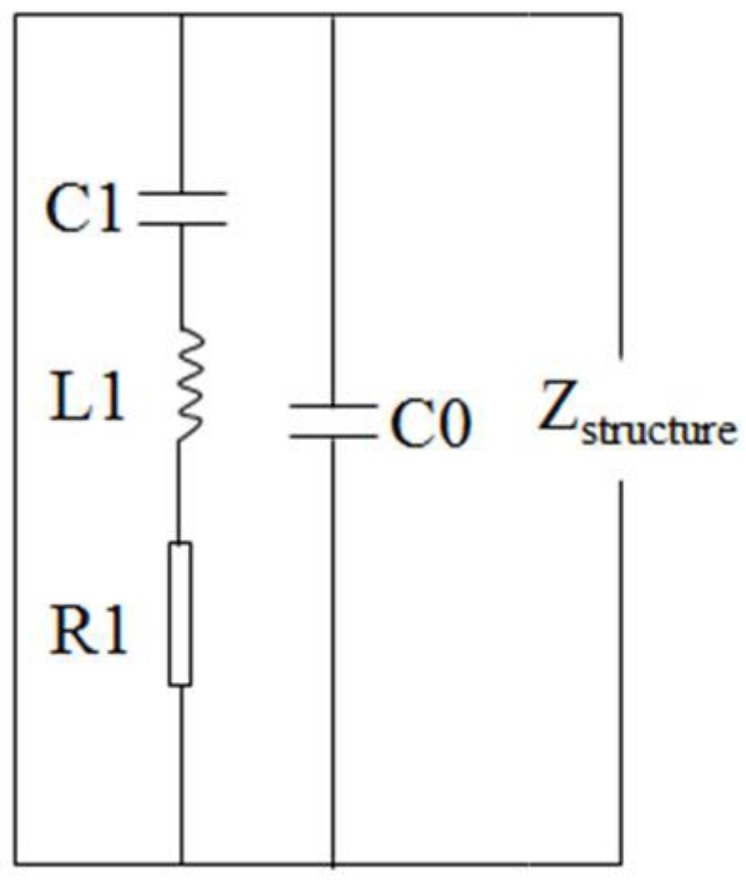
Equivalent circuit of piezoelectric materials around the resonant frequency. *C*_0_: the parallel capacitance; *R*_1_: dynamic electric resistivity; *C*_1_: dynamic electric capacitance; *L*_1_: dynamic electric inductance

The variation of resonant peaks shows that the packaging layer has influence on impedance spectra of the PZT transducer. The basic equivalent circuit of piezoelectric material around the resonant frequency is illustrated in Figure 5 [24,26].

In Figure 5, the dynamic electric parameters are consisted of resistivity *R*_1_, capacitance *C*_1_ and inductance *L*_1_, which are associated with the coupled load (i.e., packaging layer). The resonant ability of the PZT transducer weakens under the effects of mechanical damping of the packaging layer, and thus the electric impedance of the transducer changes correspondingly.

### 3.2. Impedance Spectra of PZT Transducers under Different Relative Mass Losses

Through comparing the resistance versus frequency spectra in different frequency intervals, it can be found that the resonant peaks of PZT transducers appear mainly in the frequency range of 20–500 kHz. In this study, the typical resistance versus frequency spectra in two different frequency intervals for both PZT transducers were analyzed.

**Figure 6:**
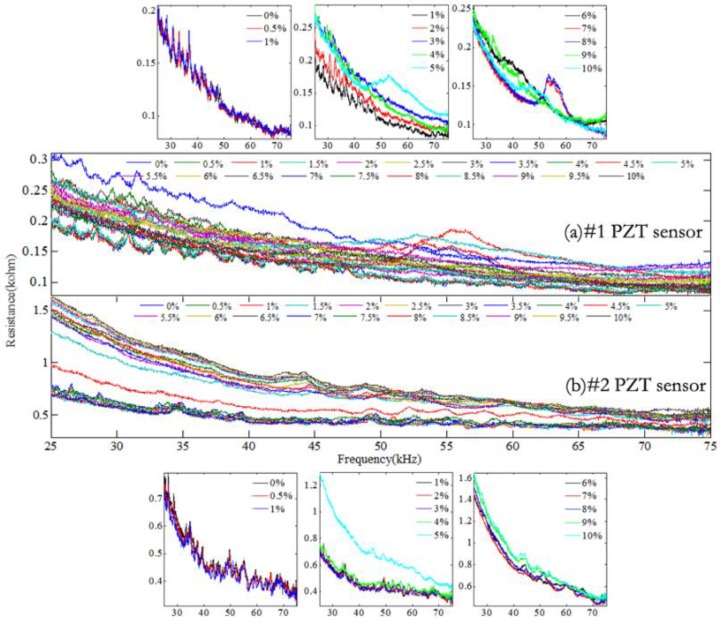
Resistance vs. frequency spectra for PZT transducers in the frequency range of 25–75 kHz.

Figure 6 shows the resistance versus frequency spectra of both PZT transducers in a frequency range of 25–75 kHz. As the relative mass loss increases, it can be observed that the resistance versus frequency curves show obvious variation, such as drifts of resonant frequency, amplitude variation of resistance, and variation in amounts of the resonant peaks. When the relative mass loss is less than 1%, the variation of resistance spectra for both PZT transducers is not obvious; that is, the weak corrosion just causes a slight resistance variation. When the relative mass loss is higher than 1%, the resistance value and the resonant frequency of the spectra have obvious variation. As the relative mass loss increases, some new resonant peaks can also be clearly observed, especially in the spectra of PZT transducer #1.

**Figure 7:**
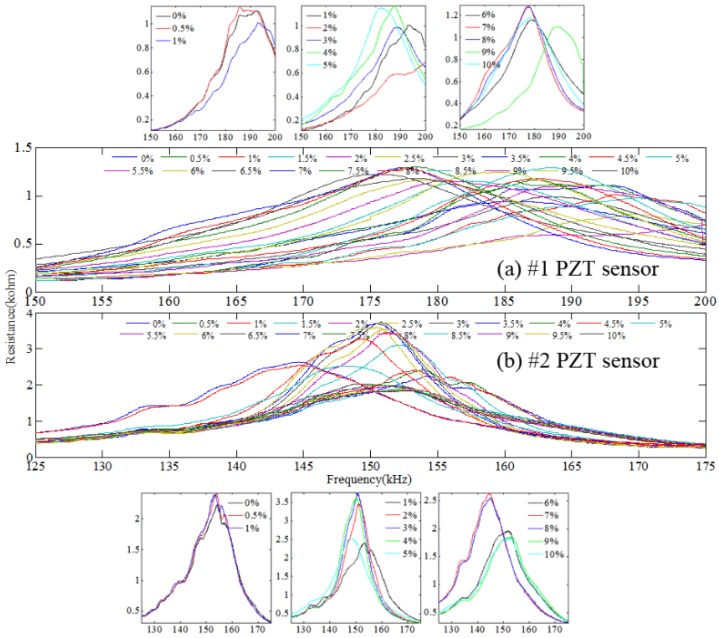
Resistance vs. frequency spectra of PZT transducers in different frequency intervals.

The dominant resonant frequencies of PZT transducers #1 and #2 appear in the frequency range of 150–200 kHz and 125–175 kHz, respectively. The resistance versus frequency spectra of both PZT transducers in this frequency interval are shown in Figure 7. It can be seen that the dominant resonant peaks of both PZT transducers shift obviously with increasing the relative mass loss. Compared with Figure 6, the variation of the resistance curve is more obvious as the relative mass loss increases, even though the relative mass loss is small.

Through investigating the resistance versus frequency spectra, the conclusion can be drawn that the spectra are greatly influenced by the mechanical impedance variation of corroded steel. As the relative mass loss increases, the frequency of the resonant peaks shifts and the amplitude of the resonant peak changes. Compared with Figure 6, the resistance versus frequency spectra in Figure 7 which contains the dominant resonant peaks of PZT transducers has a better ability to identify the initial corrosion of reinforced concrete.

### 3.3. Statistical Analysis of Impedance Spectra

The corrosion variation of steel in concrete can be observed through the resistance versus frequency spectra of PZT transducers; however, the variation law of the corrosion process cannot be obtained intuitively from the spectra. Therefore, appropriate quantitative damage indices are important to evaluate the corrosion process of reinforcement concrete. Here, the statistical method was used to analyze the variation of resistance versus frequency spectra of PZT transducers. The mathematical models of root mean square deviation (RMSD), mean absolute percentage deviation (MAPD), and correlation coefficient (CC) were established as the corrosion indices.

(6) $$\mathrm{CC}=\frac{1}{n\sigma_{R_{i}^{0}}\sigma_{R_{i}}}\sum_{i=1}^{n}\left(R_{i}^{0}-\bar{R_{i}^{0}}\right)(R_{i}-\bar{R_{i}})$$

(7) $$RMSD=\sqrt{\frac{\sum_{i=1}^{n}{\left[\left|R_{i}\right|-\left|R_{i}^{0}\right|\right]}^{2}}{\sum_{i=1}^{n}{\left[\left|R_{i}^{0}\right|\right]}^{2}}}$$

(8) $$\mathrm{MAPD}=\frac{1}{n}\sum_{i=1}^{n}\left|\frac{R_{i}-R_{i}^{0}}{R_{i}^{0}}\right|$$

where |R_i_| is the resistance value of PZT transducers in the spectra, |*R*_i_^0^| is the reference resistance value of PZT transducers when the relative mass loss is 0%, and *n* is the frequency count in the spectra.

**Figure 8:**
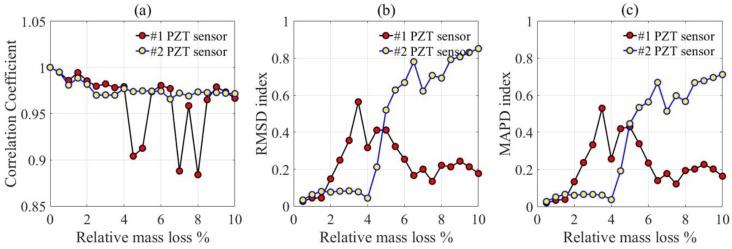
Corrosion indices of PZT transducers in a frequency interval of 25–75 kHz. (**a**) Correlation Coefficient; (**b**) RMSD index; (**c**) MAPD index. RMSD: root mean square deviation; MAPD: mean absolute percentage deviation.

The corrosion indices were calculated based on the resistance versus frequency spectra of PZT transducers in Figure 6, as shown in Figure 8. It can be observed that when the relative mass loss is less than 4%, the CC index presents a slow downward trend, but the RMSD and MAPD indices show the increasing trend, especially for PZT transducer #1. A transition exists at a relative mass loss of about 2%, and as the relative mass loss increases, the CC index decreases evidently and the RMSD and MAPD indices increase remarkably. When the relative mass loss is higher than 4%, the CC index of PZT transducer #1 shows a fluctuating variation, but that of PZT transducer #2 shows a decreasing trend. The RMSD and MAPD indices have a similar variation for PZT transducer #1, which decrease evidently as the relative mass loss increases. On the contrary, the RMSD and MAPD indices of PZT transducer #2 increase greatly as the relative mass loss increases.

**Figure 9:**
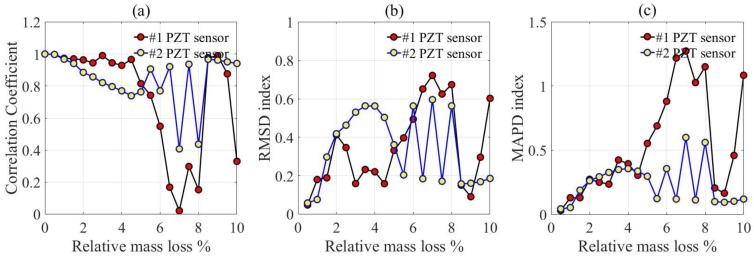
Corrosion indices of PZT transducers in a frequency interval of 150–200 kHz for transducer #1 and 125–175 kHz for transducer #2. (**a**) Correlation Coefficient; (**b**) RMSD index; (**c**) MAPD index.

In addition, the corrosion indices were also calculated based on the resistance versus frequency spectra of PZT transducers in Figure 7, as shown in Figure 9. It can be seen that when the relative mass loss is less than 4%, the CC index decreases gradually as the relative mass loss increases, while the RMSD and MAPD indices show an increasing trend, especially for PZT transducer #2. A transition also exists at a relative mass loss about 2%. When the relative mass loss is higher than 4%, all the corrosion indices show obvious fluctuation. This phenomenon indicates that there has been a violent corrosion reaction of the steel in the concrete; therefore, the resistance of PZT transducers changes greatly because of variation of the mechanical impedance of the corroded steel.

Based on the corrosion indices, the corrosion behavior of the reinforcement concrete can be categorized into the following periods: Firstly, the initial corrosion period when the relative mass loss is less than 2%. There is a smooth variation of corrosion indices in this period. Secondly, the increasing corrosion period when the relative mass loss is between 2% and 4%. There is an obvious variation of corrosion indices in this period. Finally, the rapidly developing period when the relative mass loss is higher than 4%; obvious fluctuation of corrosion indices is exhibited in this period. 

## 4. Conclusions

PZT ceramic was used as a piezoelectric element, and a mixture of cement, epoxy resin, and hardening agent was used as an encapsulating material to fabricate the embedded piezoelectric transducers. The piezoelectric impedance technique was employed to evaluate the corrosion process of reinforced concrete. As the relative mass loss increases, the resistance spectra of the piezoelectric transducers show obvious variation. The root mean square deviation (RMSD), mean absolute percentage deviation (MAPD), and correlation coefficient (CC) were established to evaluate the corrosion process of reinforced concrete, and three corrosion periods can be concluded based on these parameters; that is, the initial corrosion period when the relative mass loss is less than 2%, the increasing corrosion period when the relative mass loss is about 2–4%, and the rapidly developing period when the relative mass loss is higher than 4%.
